# Correlation between plasma glutathione peroxidase 4 and N-acetylneuraminic acid levels with clinical risk stratification and prognosis of patients with acute coronary syndrome

**DOI:** 10.15537/smj.2022.43.10.20220444

**Published:** 2022-10

**Authors:** Miao-Nan Li, Bing-Wei Bao, Ding Si-Yu, Ji Chun-Fei, Shi Xiao-Jun, Gao Da-Sheng, Gao Qin, Wang Hong-Ju

**Affiliations:** *From the Department of Cardiovascular Disease (Li, Bao, Ding, Ji, Shi, D-S. Gao, Q. Gao, Wang), The First Affiliated Hospital of Bengbu Medical College, and from the Department of Physiology (Q. Gao), Key Laboratory of Cardiovascular and Cerebrovascular Diseases, Bengbu Medical College, Bengbu, China.*

**Keywords:** acute coronary syndrome, N-acetyl-neuraminic acid, plasma glutathione peroxidase 4, plasma TIMI risk score, prognosis

## Abstract

**Objectives:**

To investigate the correlation between plasma glutathione peroxidase 4 (GPX4) and N-acetyl-neuraminic acid (Neu5Ac) with clinical risk stratification and outcomes of acute coronary syndrome (ACS) patients.

**Methods:**

Between October 2018 and July 2019, 413 patients that were scheduled for coronary angiography were enrolled in this prospective study at the First Affiliated Hospital of Bengbu Medical College, Bengbu, China. Patients were divided into control and ACS groups. Patients with ACS were divided into 3 risk levels based on their thrombolysis in myocardial infarction risk score. After discharge, ACS patients were followed for the incidence of major adverse cardiac events (MACEs). For the analysis of cumulative endpoint event occurrences, the Kaplan-Meier method was applied.

**Results:**

The ACS group had lower plasma GPX4 but higher Neu5Ac levels than the control group. There was a greater increase in plasma Neu5Ac in the high-risk group when compared with the medium-risk and low-risk groups, while GPX4 levels were higher in the low-risk group. The MACEs group had higher plasma Neu5Ac but lower GPX4 levels than the non-MACEs group. The plasma Neu5Ac was an independent risk factor but GPX4 was a protective factor for MACEs.

**Conclusion:**

Glutathione peroxidase 4 and Neu5Ac levels in plasma can be used to diagnose, stratify risks, and predict long-term outcomes in patients with ACS.

**I**n the global population, the acute coronary syndrome (ACS) is a leading cause of death and morbidity. Clinically, ACS can be divided into 3 types: I) unstable angina pectoris (UAP), ST-segment elevation myocardial infarction (STEMI), and non-STEMI.^1^ Because ACS has a heavy economic burden on families and society, it is imperative to identify factors that might help with diagnosis, prediction, or both of the risk and prognosis of ACS patients. To achieve this, it is important to understand the etiology of ACS. Although a significant amount of research has been carried out to decipher the molecular basis that underlies the pathogenesis of ACS, the exact mechanisms remain unknown. However, it is accepted that the mechanisms that lead to the pathogenesis of ACS are multifactorial, including metabolic abnormality.^2,3^ Additionally, new research indicates that iron death (ferroptosis), a new form of cell death, plays an important role in cardiovascular disease. For instance, targeted intervention of iron death could effectively prevent and treat heart diseases.^4-6^

Selenium-dependent glutathione peroxidase 4 (GPX4) is a member of the GPX family and protects cells from membrane lipid oxidation-linked damage.^7^ Glutathione peroxidase 4 is antioxidative, which makes it effective at preventing and treating a variety of tissue injuries and diseases.^8-10^ Several studies have linked ferroptosis to the deactivation of GPX4, which then causes the accumulation of reactive oxygen radicals on membrane lipids.^3,11^ In addition, GPX4 can alleviate inflammation.^12^ Researchers have found that decreased levels of GPX1 are associated with higher cardiovascular risk.^13^ However, another study showed that the circulating levels of GPX factors, including GPX4, were significantly enhanced in ACS patients compared to controls, and attributed this increase to the bodies response to oxidative stress during ACS.^14^ Therefore, how the plasma GPX4 levels change exactly in ACS patients and what roles GPX4 has in the initiation and progression of ACS need to be determined.

Numerous glycoproteins, glycopeptides, and glycolipids contain N-acetyl neuraminic acid (Neu5Ac) as their basic component, and the Neu5Ac protein serves a wide range of biological functions and plays an important role in cancer, for example.^15^ In addition, Neu5Ac is involved in heart diseases. Serum Neu5Ac levels are linked to atrial fibrillation and play a key role in human acute myocardial infarction.^16^ These observations support the theory that high circulating levels of Neu5Ac might contribute to the development of heart diseases. Our group recently reported that increased serum Neu5Ac levels were related to injury of cardiomyocytes in patients with ACS.^17^ However, whether the circulating levels of Neu5Ac and GPX4 were related to the clinical outcomes of ACS remains unknown. Therefore, this study was carried out to evaluate the correlation between plasma GPX4 and Neu5Ac levels with thrombolysis in myocardial infarction (TIMI) risk score and the prognosis of ACS patients.

## Methods

This single-center prospective observational study enrolled 413 (240 males and 173 females) ACS patients (aged 62.2±10.9 years) who were scheduled to undergo coronary angiography at the First Affiliated Hospital of Bengbu Medical College, Bengbu, China, between October 2018 and July 2019. Patients that had one of the following ACS, which included: I) UAP; II) STEMI, and III) non-STEMI, were included in this study.^18,19^ The results of coronary angiography were interpreted according to the criteria recommended by the 2001 American College of Cardiology (ACC)/American Heart Association (AHA).^20^ The control group included patients that underwent coronary angiography in the hospital over the same period without ACS. This study excluded patients with any of the following conditions: I) severe liver and kidney dysfunction; II) hematopoietic diseases; III) infectious diseases; IV) tumors; or V) other wasting diseases. Based on the inclusion and exclusion criteria, the control group had 108 patients and the ACS group had 305 patients.

An informed consent form was completed by all participants before being enrolled in the study, and approval from the First Affiliated Hospital of Bengbu Medical College, Bengbu, China, was obtained (approval number: BYYFY-2018KY23).

Smoking in this study was defined as a person who has smoked continuously ≥1 cigarette per day for >1 year.^21^ The Diagnostic Criteria in Diabetes-2021 were used to diagnose diabetes.^22^ The definition of hypertension was a systolic blood pressure over 140 mm Hg or a diastolic blood pressure over 90 mm Hg after repeated measurements over time in accordance with the 2020 International Society of Hypertension Global Hypertension Practice Guidelines.^23^ Atrial fibrillation was diagnosed by the criteria proposed by the task force for the diagnosis and management of atrial fibrillation of the European Society of Cardiology.^24^

Major adverse cardiac events (MACEs): recurrent angina, heart failure, recurrent myocardial infarction, stroke, hemorrhage, revascularization, stent thrombosis, stent restenosis, cardiogenic death, and all-cause death. Re-hospitalization due to one or more of the previously mentioned reasons was counted as a MACE.

Patients were admitted to hospital early morning with an empty stomach, and 5 mL of cubital venous blood drawn into heparin- and ethylenediamine tetraacetic acid (EDTA) treated tubes. The blood samples in the heparin-treated tubes were sent to the testing center for the analysis of biochemical tests, including fast blood glucose, total cholesterol (TC), triglyceride (TG), high-density lipoprotein cholesterol (HDL-C), low-density lipoprotein cholesterol (LDL-C), D-dimer, and C-reactive protein (CRP).

To measure plasma GPX4 and Neu5Ac, blood samples in the EDTA-treated tubes were sent to the heart and lung laboratory and serum was separated within 30 minutes, stored at -80°, and used to measure plasma GPX4 and Neu5Ac levels using an enzyme-linked immunosorbent method and liquid chromatography tandem mass spectrometry.^25,26^

Coronary angiography was carried out via the Judkins method, and the results were evaluated using the 2001 ACC/AHA report for the management of cardiovascular diseases.^20,27^ The angiography showed that there was coronary artery stenosis ≥70% for coronary stent implantation and that a drug-eluting stent was implanted in the lesion in patients. Individual patient’s coronary angiographic results and stent implantation process were recorded. The success criteria for stent implantation were based on international practice, for example, residual stenosis ≤20%, and a TIMI3 blood flow. The Gensini score was used to quantitatively calculate the degree of stenosis for each diseased vessel, which was independently evaluated by 2 cardiologists, and the average data were calculated.^28^

The clinical risk score for TIMI risk score was used to stratify patients in the UAP and acute myocardial infarction groups, and patients with UAP, non-STEMI, and STEMI were stratified according to different scoring standards.^29,30^

All patients were monitored during hospitalization. An outpatient clinic or telephone follow-up was carried out monthly for 15 months following discharge for ACS patients with MACEs.^31^ Four patients were lost to follow-up. Major adverse cardiac events in this study were defined as ≥1 of the following: I) recurring chest pain; II) heart failure; III) stroke; IV) recurring myocardial infarction; V) hemorrhage; VI) revascularization; VII) stent thrombosis; VIII) restenosis in the stent; and IX) death.^31^ Based on the presence or absence of MACEs, ACS patients were divided into the MACEs group (n=37) and the non-MACEs group (n=268).

Research and outcomes were not developed with the involvement of patients or the public. We aim to publish the study results as open access, which will be readily available to the public.

### Statistical analysis

All analyses were carried out using the Statistical Package for the Social Sciences, version 21.0 (IBM Corp., Armonk, NY, USA). Measured data are presented as mean ± standard deviation (SD). Data that are normally distributed were analyzed with variance analysis, whereas data that are not normally distributed were analyzed using non-parametric tests. Comparing data between the 2 groups was accomplished by using the Student’s T-test; describing the classification data was accomplished by using the composition ratio and comparing it by using the Chi-squared test. The correlation analysis was carried out using a bivariate correlation analysis. The risk factors for MACEs were determined using Cox’s risk proportional regression model, and Kaplan-Meier was used to illustrate the curve of endpoints, which included recurrent angina pectoris, heart failure, recurrent myocardial infarction, stroke, hemorrhage, revascularization, stent thrombosis, stent restenosis, cardiogenic death, and all-cause death. *P*-values of <0.05 indicate a significant difference.

## Results

This study included 413 patients, who were divided into a control group (n=108; age: 57.01±9.84 years; 43 males and 65 females) and an ACS group (n=305; age: 63.88±10.76 years; 197 males and 108 females). Patients in these 2 groups were compared in terms of demographics and clinical characteristics. As listed in Table 1, no significant differences were observed for TC, TG, AF, HDL-C, LDL-C, D-dimer, and CRP (*p*>0.05). However, the ACS group had older patients and more males and smokers than the control group (*p*<0.01). In addition, the ACS group had a higher diabetic rate, higher levels of blood uric acid, blood sugar, blood creatinine, and high-density lipoprotein (*p*<0.01). In addition, the ACS group had lower levels of plasma GPX4 but higher levels of plasma Neu5Ac than the control group (p<0.05).

**Figure 1:**
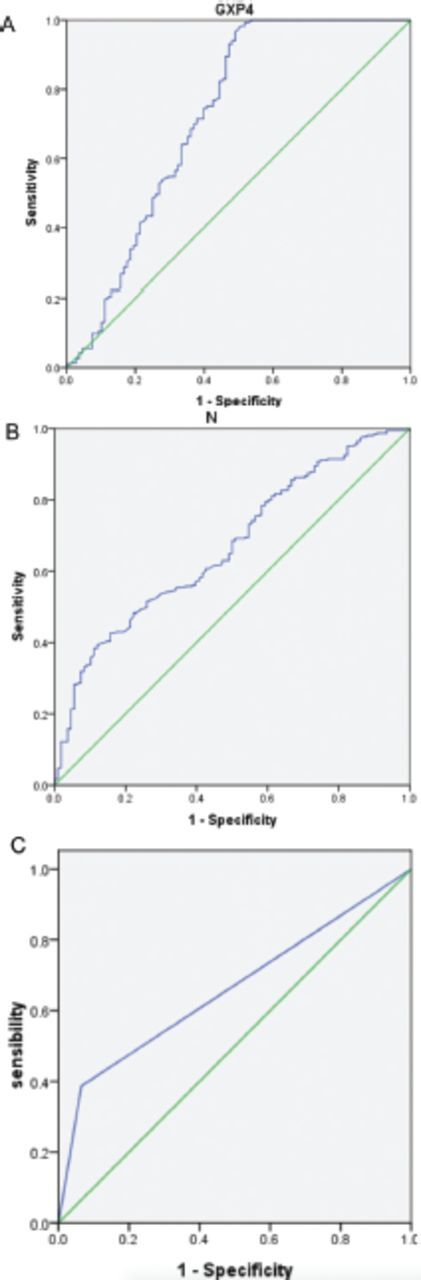
- Determination of cut-off levels for plasma glutathione peroxidase 4 (GPX4) and N-acetyl-neuraminic acid (Neu5Ac) in auxiliary diagnosis of acute coronary syndrome. A) GPX4 receiver operating characteristic (ROC) curve; B) Neu5Ac ROC curve; and C) combined GPX4 and Neu5Ac curve.

Receiver operating characteristic (ROC) curves were used to determine whether the levels of plasma GPX4 and Neu5Ac held a value in the auxiliary diagnosis of ACS. The plasma GPX4 levels were valuable in the auxiliary diagnosis of ACS (area under the curve [AUC]: 0.723 [0.657-0.790]), sensitivity of 97.7%, specificity 50.0%, and a cut-off value of 131.06 ng/mL (Figure 1A). Similarly, the ROC curve showed that the plasma Neu5Ac levels were valuable in the auxiliary diagnosis of ACS (AUC: 0.667 [0.610-0.724]), sensitivity 39.3%, specificity 88.0%, and a cut-off value of 286.50 ng/mL (Figure 1B). The cut-off values of these 2 indicators were combined as a positive group and a joint calculation of the ROC curve was carried out. This demonstrated that there was still a value for the auxiliary diagnosis of ACS (AUC: 0.661 [0.607-0.715]), sensitivity of 38.7%, specificity of 93.5% (Figure 1C). Therefore, the circulating levels of GPX4 and Neu5Ac (alone or combined) could have value in the auxiliary diagnosis of ACS.

**Figure 2:**
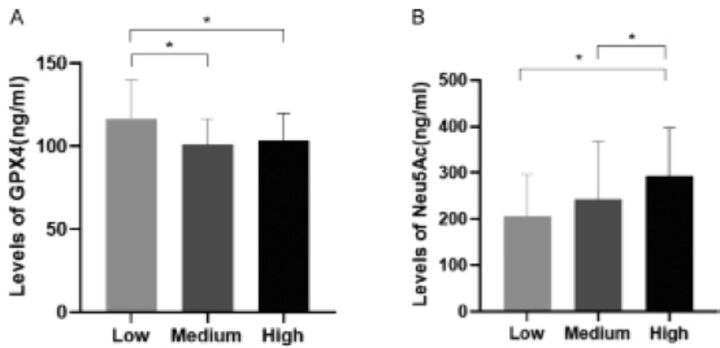
- Correlation between plasma glutathione peroxidase 4 (GPX4) and N-acetyl-neuraminic acid (Neu5Ac) levels with thrombolysis in myocardial infarction (TIMI) risk stratification: A) GPX4 is negatively correlated with TIMI risk score; B) Neu5Ac is positively correlated with TIMI risk score.

The ACS patients were further divided into 3 groups on the basis of TIMI scores: I) high-risk group (n=42); II) medium-risk group (n=221); and III) low-risk group (n=42), and the association between plasma GPX and Neu5Ac levels with TIMI risk stratification was examined. There was a significantly higher plasma level of GPX4 in the low-risk group than in the medium- and high-risk groups. Figure 2A indicated an inverse correlation between the plasma GPX4 levels with the TIMI risk stratification (*p*<0.05). Additionally, the Neu5Ac levels in high-risk patients were significantly higher than in medium- and low-risk patients, which indicated a positive correlation between Neu5Ac and TIMI risk (Figure 2B; *p*<0.05).

**Table 1:** - Comparison of demographic and baseline clinical characteristics of patients between groups.

Variables	ACS group (n=305)	Control group (n=108)	t/χ2	*P*-values
Age (years), mean±SD	63.88±10.76*	57.01±9.84	5.346	0.000
Male	197 (0.6)*	43 (0.4)	19.603	0.000
Smoker	51 (0.2)*	8 (0.1)	6.928	0.008
Hypertension	197 (0.7)	59 (0.6)	3.358	0.067
DM	77 (0.3)*	10 (0.1)	12.259	0.000
AF	14 (0.1)	5 (0.1)	0.769	0.381
Previous stroke	24 (0.1)	7 (0.1)	0.046	0.830
FBG (mmol/L)	5.16 (4.60-7.00)*	4.99 (4.57-5.81)	3.848	0.000
UA (µmol/L)	305.00 (253.00-374.00)*	284.50 (245.25-357.75)	2.252	0.025
SCr (µmol/L)	67.00 (61.00-75.00)*	64.00 (57.00-69.00)	4.635	0.000
TC (mmol/L)	3.81 (3.05-4.54)	3.54 (2.90-4.67)	0.855	0.442
TG (mmol/L)	1.35 (0.92-1.92)	1.21 (0.85-1.66)	1.336	0.182
LDL-C (mmol/L)	1.93 (1.42-2.69)	0.95 (0.79-1.17)	0.306	0.760
HDL-C (mmol/L)	0.87 (0.72-1.04)*	1.21 (0.85-1.66)	2.174	0.030
Lp(a) (mg/L)	225.00 (82.50-373.50)	224.00 (120.75-461.50)	0.819	0.413
CRP (mg/L)	1.80 (0.60-5.00)	1.40 (0.69-3.95)	0.705	0.481
D-dimer (mg/L)	0.26 (0.19-0.44)	0.23 (0.08-0.39)	0.524	0.600
GPX4 (ng/mL)	104.39 (89.12-115.38)*	130.98 (98.27-148.56)	7.449	0.000
Neu5Ac (ng/mL)	250.00 (130.67-318.50)*	239.50 (111.48-285.75)	2.366	0.018

Values are presented as a number and precentage (%) and median (minimum-maximum). *p-value of <0.05 versus control. ACS: acute coronary syndrome, DM: diabetes mellitus, AF: atrial fibrillation, FBG: fasting blood glucose, UA: uric acid, SCr: serum creatinine, TC: total cholesterol, TG: triglyceride, LDL-C: low-density lipoprotein cholesterol, HDL-C: high-density lipoprotein cholesterol, Lp(a): lipoprotein(a), CRP: C-reactive protein, GPX4: glutathione peroxidase 4, Neu5Ac: N-acetylneuraminic acid, SD: standard deviation

Telephone or outpatient visits were used to follow up with patients in the ACS group for an average of 15 months after discharge, and 4 of them were lost. The MACEs were developed in 37 patients. The correlation between plasma GPX4 and Neu5Ac levels with the occurrence of MACEs in ACS was examined. The MACEs group had significantly lower plasma GPX4 levels than the non-MACEs group (92.66 [82.78-105.11] versus 106.29 [92.15-121.63] ng/mL; *p*<0.05), but had significantly higher plasma Neu5Ac levels than the non-MACEs group (270.00 [134.93-340.69] versus 247.50 [123.54-313.50] ng/mL; *p*<0.05). In addition, Cox’s regression analysis was used to retrieve the risk factors for MACEs. As listed in Table 2, hypertension, HDL-C, DM, and atrial fibrillation were not risk factors for MACEs. However, plasma Neu5Ac and TC levels were independent risk factors for MACEs and plasma GPX4 levels were a protective factor for MACEs (Table 2).

**Table 2:** - Determination of risk factors for major adverse cardiac events by COX regression analysis.

Variables	B	OR (95% CI)	*P*-values
GPX4 (ng/mL)	-0.24	0.976 (0.955-0.997)	0.026
Neu5Ac (ng/mL)	0.001	1.001 (1.000-1.002)	0.003
TC (mmol/L)	0.383	1.466 (1.084-1.982)	0.013
HDL-C (mmol/L)	-0.966	0.381 (0.073-1.977)	0.250
Hypertension	0.030	1.031 (0.452-2.349)	0.943
DM	0.138	1.148 (0.467-2.821)	0.763
AF	0.169	1.184 (0.154-9.122)	0.871

OR: odds ratio, CI: confidence interval, GPX4: glutathione peroxidase 4, Neu5Ac: N-acetylneuraminic acid, TC: total cholesterol, HDL-C: high-density lipoprotein cholesterol, DM: diabetes mellitus, AF: atrial fibrillation

**Figure 3:**
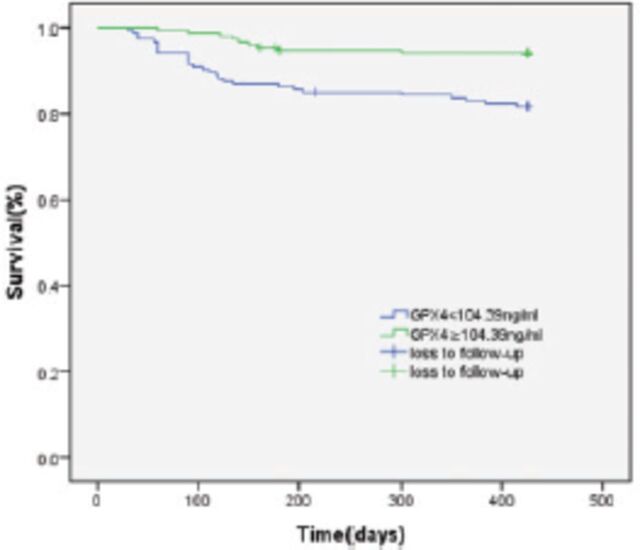
- Predictive value of glutathione peroxidase 4 and N-acetyl-neuraminic acid for long-term endpoint events in acute coronary syndrome patients. GPX4: glutathione peroxidase 4, Neu5Ac: N-acetyl-neuraminic acid

The ability of plasma GPX4 and Neu5Ac to predict the long-term endpoint events in ACS patients was investigated. Medians of GPX4 (104.39 ng/mL) and Neu5Ac (250 ng/mL) were used as the cut-offs. In total, 9 out of 149 patients in the GPX4 ≥104.39 ng/mL group had an endpoint event and 28 out of 156 patients in the GPX4 <104.39 ng/mL group had an endpoint event during follow. The average time for the occurrence of endpoint event between these 2 groups was 408.59 days and 373.36 days, and the log-rank test yielded χ^2^=11.091, (*p*<0.05; Figure 3). In the Neu5Ac groups, there was no significant difference in the average days for an endpoint event to occur and the number of patients who experienced an endpoint event (*p*>0.05).

## Discussion

In this study, the correlations between plasma GPX4 and Neu5Ac with the clinical risk stratification and prognosis of ACS patients were investigated. The major findings from this study were: I) ACS patients had significantly lower plasma GPX4 levels but higher plasma Neu5Ac levels than the control subjects; II) GPX4 had a negative correlation but Neu5Ac had a positive correlation with the TIMI risk stratification; III) plasma Neu5Ac may be independent risk factor for the incidence of MACEs; however, plasma GPX4 was a protective factor for MACEs; IV) plasma GPX4, or Neu5Ac, or a combination of them, had a value in the auxiliary diagnosis of ACS; and V) plasma GPX4 had a value to predict the prognosis of ACS.

Discovering the biomarkers for the diagnosis and prognosis of ACS has received extensive research. Currently, a number of biomarkers, including cardiac troponin I, creatine kinase MB isoform (CK-MB), and creatine kinase, have been used in clinics.^32^ In this study, the potential of plasma GPX4 and Neu5Ac to serve as potential biomarkers for the risk stratification and prognosis of ACS were assessed. The findings revealed that ACS patients had lower plasma GPX4 levels than control subjects, which indicated that plasma GPX4 had a negative correlation with ACS. A previous study showed that GPX4 was significantly upregulated in ACS patients compared with control patients, and another study suggested that lower activity of GPX was associated with increased risk of cardiovascular diseases.^13,14^ This study suggested that lower circulating levels of GPX4 were observed in ACS patients. The reason for these findings was not clear; however, patient selection and different diagnostic criteria could have had an impact. Because GPX4 exhibits antioxidative activity, and oxidative stress has an important role in the pathogenesis of ACS, the decreased activity of GPX4 could have detrimental effects on the cardiovascular system and increased activity of GPX4 improves it.^33^ In addition, the ROC analysis suggested that the circulating levels of GPX4 might be valuable in the auxiliary diagnosis of ACS with sensitivity of 97.7%, specificity of 50.0%, and a cut-off value of 131.06 ng/mL. Mechanistically, GPX4 might provide beneficial effects for ACS patients through a number of mechanisms, including antioxidative activity, and rebalancing iron metabolism to reduce ferroptosis.^34-36^

In this study, the circulating levels of Neu5Ac, a family of monosaccharides with a 9-carbon backbone, were found higher in ACS patients than controls, which suggested that plasma Neu5Ac was positively correlated with ACS.^37^ Metabolomics in cardiovascular diseases has received significant attention and might provide early diagnosis, intervention, and prognosis for ACS.^38,39^ Current research suggests that plasma Neu5Ac might promote atherosclerosis, which agrees with the results of this study.^37^ Mechanistically, Neu5Ac negatively affects the cardiovascular system through inflammation, interference with iron metabolism, and the promotion of platelet thrombosis and has been suggested as a target for prevention of atherosclerosis.^40^ In this study, the ROC analysis suggested that the circulating levels of Neu5Ac were valuable in the auxiliary diagnosis of ACS (AUC: 0.667 [0.610-0.724]), with sensitivity of 39.3%, specificity of 88.0%, and a cut-off value of 286.50 ng/mL.

In this study, GPX4 or Neu5Ac could serve as a potential biomarker for ACS diagnosis. A combination of them could have value in the auxiliary diagnosis of ACS. However, which one was superior was not determined in this study and this could be the subject of future research.

Based on the previous observations, the correlation between plasma GPX4 and Neu5Ac levels and TIMI risk score, which is a widely used scoring system to stratify the risk of ACS patients, was examined.^29^ The plasma Neu5Ac levels in the high-risk group were significantly higher than those in the medium- and low-risk groups, which suggested that the plasma Neu5Ac levels were positively associated with high-risk ACS patients. However, the plasma GPX4 levels in the low-risk group were significantly higher than the medium- and high-risk groups, which suggested that the plasma GPX4 levels were negatively associated with the high-risk ACS patients. In combination, these observations indicate that the levels of plasma GPX4 and Neu5Ac might be used as indicators for the TIMI risk stratification of ACS patients.

The patients in the ACS group were followed-up for an average of 15 months. Based on the previous observations, the MACEs group had significantly higher plasma Neu5Ac levels but lower plasma GPX4 levels than the non-MACEs group. Cox regression analysis showed that plasma Neu5Ac and TC levels were independent risk factors for MACEs; however, GPX4 was a protective factor for MACEs. In addition, the average time of the endpoint event in the GPX4 <104.39 ng/mL group was earlier than that in the GPX4 ≥104.39 ng/mL group, which indicated that GPX4 could be used as a predictor for the prognosis of ACS patients. Of interest, Neu5Ac did not demonstrate this function in this study.

### Study limitations

First, this study was a single-center observational study with relatively small sample size. Second, ACS patients with severe liver and kidney insufficiency and cardiopulmonary insufficiency were excluded from this study, which was probably linked to the low incidence of MACEs that were observed in this study. Third, the patients in this study were heterogenous. In addition, the potential differences in dietary sources of Neu5Ac among the included patients might have affected the blood circulating levels of Neu5Ac, which might be a confounding factor of this study. Fourth, the follow-up period was short; therefore, the long-term association between plasma GPX4 and Neu5Ac levels for the prognosis of ACS patients requires further investigation. Finally, the efficacy of both biomarkers with the currently used clinical used biomarkers for ACS diagnosis in this study was not compared.

In conclusion, plasma GPX4 and Neu5Ac levels are associated with the clinical risk stratification of ACS patients and could have value for the auxiliary diagnosis and prognostic prediction of ACS patients. Therefore, plasma GPX4 and Neu5Ac levels could offer valuable guidance for the clinical application and targeted prevention and treatment of ACS.
